# Learning from a Learning Collaboration: The CORC Approach to Combining Research, Evaluation and Practice in Child Mental Health

**DOI:** 10.1007/s10488-014-0592-y

**Published:** 2014-09-19

**Authors:** Isobel Fleming, Melanie Jones, Jenna Bradley, Miranda Wolpert

**Affiliations:** 1 Child Outcomes Research Consortium, 21 Maresfield Gardens, London, NW3 5SU UK; 2 Evidence Based Practice Unit, London, UK

**Keywords:** Learning collaboration, Routine outcome monitoring, CORC, PROMS and PREMS

## Abstract

This paper outlines the experience of the Child Outcomes Research Consortium—formerly known as the CAMHS Outcomes Research Consortium; the named changed in 2014 in recognition of the widening scope of the work of the collaboration; a learning collaboration of service providers, funders, service user groups and researchers across the UK and beyond, jointly committed to collecting and using routinely collected outcome data to improve and enhance service provision and improve understanding of how best to help young people with mental health issues and their families.

## Context

The Child Outcomes Research Consortium (CORC—formerly known as the CAMHS Outcomes Research Consortium; the named changed in 2014 in recognition of the widening scope of the work of the collaboration) was formed in 2002 by a group of child mental health clinicians, managers and funders all working in the National Health Service (NHS) in England. They worked across five different service providers across the country, but shared a mutual curiosity as to the effectiveness of their and their colleagues’ practice and how best to improve their own practice.

They determined that one way to find out about the impact of their work was to ask those they worked with (not routine practice then or now) and thus set about exploring appropriate tools to try to access these views in a systematic way (Wolpert et al. 2012). Interest grew amongst other services and interested academics joined the founding group. The collaboration opened to wider membership in 2004 and was formalised as a not-for-profit learning consortium in 2008 (see www.corc.uk.net).

Over the last decade the collaboration has grown to include over half of all services across the UK (70 membership groupings) with members also in Scandinavia and Australia, and seeks to act as a peer-learning group (Fullan 2009). It also increasingly includes a range of voluntary sector and counselling services.

The collaboration has pioneered the routine use of patient-reported outcome and experience measures (PROMs and PREMs) across child mental health services in England (supported by research reviewed elsewhere in this special issue) and has informed and contributed to policy development (Department of Health 2004, 2012). Its work and learning has underpinned the current national service transformation initiative: children and young people’s improving access to psychological therapies (CYP IAPT; http://www.cypiapt.org/) which seeks to implement patient-reported routine outcome measurement across children’s mental health services in England.

The Child Outcomes Research Consortium has recently introduced a self-review and accreditation system to allow members to internally assess quality and gain external assurance that they are implementing best practice in outcome evaluation.

From the outset, CORC has sought to bridge the worlds of clinical decision-making, evaluation and research. Table 1 offers a conceptualisation of the way that the collaboration conceived this continuum and outlines the role of CORC at each level.

**Table 1:** CORC support for clinical practice, service evaluation and research

Aspect	Primary aim	How CORC supports each aim
Clinical practice	Aid clinical decision making	• Makes measures freely available
		• Trains clinicians in use and interpretation of measures, UPROMISE and bespoke trainings
		• Advises on how to choose data collection systems
		• Provides access to free data collection systems
Service evaluation	Support performance management	• Provides team and service level reports that compare service with others using appropriate metric
		• Provides advice on how to consider such data collaboratively using the MINDFUL approach
		• Present reports at service meetings
Research	Contribute to the evidence base	• Analyses collated data to support member enquiries
		• Used data to answer key questions
		• Shares findings with members and publically as relevant
		• Submits to articles to peer review journals and publishes findings

This is a challenging agenda and there are clear tensions, as well as interdependencies, between the desire to use outcomes to directly inform clinical practice and using them to inform research and service evaluation (Wolpert 2014). Below we elaborate the key challenges faced in trying to use patient-reported routine outcome and experience measurement to contribute to research, evaluation and practice, and how CORC has tried to address them. In this paper we are reflecting on the practical issues and sustainability, rather than implementation (see CORE paper for a methodological approach) of CORC methodologies.

## PROMs, PREMs and Clinical Practice

The Child Outcomes Research Consortium emphasises that any feedback measure should be used in the context of collaborative working and with an aspiration to shared decision-making to directly inform clinical work (Law 2012; Law and Wolpert 2014). Practitioners are encouraged to consider the outcomes of clients they see using normative data and to discuss this in supervision (Law and Wolpert 2014). This approach is supported by service users themselves (Roberson 2011).

It should be noted that the collaboration has not yet finalised ways to support members to track progress for individual clients against trajectories of change. This is something that the collaboration is seeking to pursue: learning from the approach pioneered by Lambert, Bickman, Duncan, Miller and others,work is underway to develop trajectories of change using a range of measure for a UK population.

As reported elsewhere in this special issue, there are well-recognised challenges to encouraging clinicians to use such measures as part of their routine practice including: a) concerns about inappropriate use and impact on therapeutic relationship; b) lack of confidence in choosing and using measures; c) concerns about insufficient support for increased administrative demands to inadequate data systems to support the collection of considerable amounts of additional data fields (Badham 2011; Curtis-Tyler 2011; de Jong et al. 2012; Johnston and Gowers 2005; Moran et al. 2012; O’Herlihy 2013; Wolpert 2013.)

The collaboration addresses these challenges as follows:

- In terms of concerns about impact on the therapeutic relationship; CORC explicitly recognises the dangers of forms being used as a “tickbox exercise” without regard for the therapeutic relationship (Wolpert 2014). CORC stresses there may be a necessary stage of “feeling clunky” that clinicians have to work through (Abrines et al. 2014) and recommends considering starting small with a few clinical staff so as to have the opportunity to “work through the bumps” in the processes (Edmondson et al. 2001).
- In terms of concerns arising from lack of confidence; CORC provides a range of free support materials on the website, including video training materials for both clinicians and supervisors (http://www.corc.uk.net/resources/implementation-support/training-videos/). Specialist one-and three-day training courses (U-PROMISE) has been developed by CORC in collaboration with others to ensure that clinicians and supervisors can use the tools effectively. This training has been shown to increase clinicians’ positive attitudes to and self-efficacy when using PROMs and feedback (Edbrooke-Childs et al. 2014).
- In terms of insufficient resources and support to allow for data collection, CORC provides guidance to funders of the need to resource and support this activity (http://www.corc.uk.net/wp-content/uploads/2012/03/CORCs-Position-on-CQUIN-targets-03042013.pdf) and also provides free databases to members to try to support them whilst their services find the best ways to collect the data routinely (http://www.corc.uk.net/resources/implementation-support/databases-templates-and-info-to-send-to-corc/).

## PROMs, PREMs and Service Evaluation

Collaborating services send their data to a central team of researchers and data analysts who produce reports that allow comparison with relevant comparators. A dashboard is being trialled to allow for a rapid review of key data. These reports are tailored to members’ needs in relation to four main domains of service metrics: 1) Who is my service seeing; 2) How well are we addressing their needs; 3) What do service users think of their support; 4) How good is our evidence on what we are doing and what could we be doing better?

Members are also offered bespoke reporting in more depth, which includes statistical comparisons of service outcomes with those of other services using funnel plots and other relevant visual representation.

Members are encouraged to use these reports to consider their outcomes in comparison with others, to inform discussions with commissioners and others in line with practice-based evidence (Wolpert et al. 2014). CORC recommends a systematic and collaborative approach to consideration of such data by service providers, funders and users adopting the ‘MINDFUL’ framework, whereby appropriate statistical comparisons are made in relation to the most meaningful clinical unit (in the UK this is the multidisciplinary team) employing multiple perspectives and harnessing the strength of a learning collaboration (Wolpert et al. 2014).

This MINDFUL framework (see Box 1) involves: a consideration of multiple perspectives, interpreting differences in the light of the current base of evidence, a focus on negative differences when triangulated with other data, directed discussions based on ‘what if this were a true difference’ which employ the 75–25 % rule (discussed further below), the use of funnel plots as a starting point to consider outliers, the appreciation of uncertainty as a key contextual reality and the use of learning collaborations to support appropriate implementation and action strategies.

**Box 1:** The MINDFUL framework

MINDFUL approach to using data to inform performance management in teams (Wolpert et al. 2014)
• Multiple perspectives: child, parent, practitioner considered separately
• Interpretation: team or individual level or care pathway
• Negative differences: as a starting point
• Directed discussions: focus on what one would do if negative differences were real (75 % discussion time) rather than examining reasons for why they might be not real (25 % discussion time)
• Funnel plots: a good way to present data to reduce the risk of over-interpretation but still only a starting point
• Uncertainty: important to remember that all data are flawed and that there is a need to triangulate data from a variety of sources
• Learning collaborations: CORC supports local learning collaborations of service users, commissioners and providers, to meaningfully interpret data

Key challenges to using data for service evaluation include a) data completeness b) data quality and c) inappropriate use of data.

The Child Outcomes Research Consortium has sought to respond these challenges as follows:

- In relation to data completeness, CORC collects information on how many referrals there are to a service and works with services to compare their data completeness (Mellor-Clark et al., in this issue). This remains a real challenge on a number of levels, including in terms of getting clinicians to use measures but also ensuring that data is entered on relevant systems. However, an independent audit found that the implementation of CORC protocols across a service (2011–2013) was associated with a doubling in the use of repeated outcome measurement during this period (30–60 %; Hall et al. 2013).
- In relation to data quality, data is checked back and forth between the central team and collaborating services. CORC runs implementers’ meetings every 6 months for those in charge of collecting data and has developed a learning community of data managers who are increasingly skilled in understanding issues surrounding data management. CORC has also greatly contributed to raising the awareness of the use and type of outcome measures, which is likely to have long term effects on data quality (Hall et al. 2013).
- In relation to an inappropriate use of data for performance management as part of this ‘MINDFUL’ framework, a sequenced approach to questioning the service and team-level reports is recommended, including consideration of data quality and appropriateness of tools used. The advice is for services to use funnel plots to consider variation in order to minimise the over-interpretation of random variation (Spiegelhalter 2005; Fugard et al. 2014.) It is recommended that service discussions start by considering the outliers who are performing more poorly that expected. Whilst recognising that these negative outliers may be artefacts related to data quality, it is also important to consider the possibility that they reflect real differences. To contract the human tendency to explain any negative differences as data errors, CORC promotes the spending of 25 % of discussion time on considering data quality concerns, and 75 % of time on a thought experiment to consider *if these data were showing up problems in our practice what might they be and how might we investigate this and rectify these issues* (Wolpert et al. 2014).

## PROMs, PREMs and Research

Over the last decade, CORC members have built up a rich (if flawed) dataset consisting of over a quarter of a million records (263,928 as of 24th February 2014) although only 24 % have meaningful outcome data. CORC has started to mine this data on behalf of members to answer key questions that may help inform our understanding of how best to help children and young people with mental health issues, always bearing in mind the need for caution given the missing data (Clark et al. 2008). In doing so, we are able to close the loop, turning practice-based evidence to evidenced-based practice.

The Child Outcomes Research Consortium now has a clear protocol whereby members (and non-members) can apply to use the data or request for analyses to be carried out by the central team. Key analyses already published include consideration of the sort of goals young people set for themselves when they come to therapy (Bradley et al. 2013) analysis of measure of service satisfaction (Brown et al. 2012) and analysis of service-level outcome (Wolpert et al. 2012). Further analyses currently in progress include an exploration of impact of evidence-based practice and a comparison of outcomes achieved between those seen in clinical services and those not seen in the community.

## Conclusion

Bridging the worlds of research, service evaluation and clinical decision-making remains a complex and challenging agenda. CORC certainly does not have all the answers and daily obstacles remain. We hope that by sharing our experience we can help advance further work in this challenging but worthwhile area.
